# Sustainable Poly (Methacrylic Acid)/Nanocellulose Hydrogel for Controlled Simultaneous Release of Active Substances for Skin Protection

**DOI:** 10.3390/gels11100838

**Published:** 2025-10-18

**Authors:** Katarina M. Antic, Maja D. Markovic, Vesna V. Panic, Pavle M. Spasojevic, Bojana D. Balanc, Milica M. Spasojevic Savkovic, Sanja I. Savic

**Affiliations:** 1 Innovation Centre of Faculty of Technology and Metallurgy, University of Belgrade, 11000 Belgrade, Serbia; mmarkovic@tmf.bg.ac.rs (M.D.M.); vpanic@tmf.bg.ac.rs (V.V.P.); pspasojevic@tmf.bg.ac.rs (P.M.S.); bisailovic@tmf.bg.ac.rs (B.D.B.); 2 Faculty of Technical Sciences, University of Kragujevac, 32000 Cacak, Serbia; 3 Innovative Centre of the Faculty of Chemistry, University of Belgrade, 11000 Belgrade, Serbia; smilica84@gmail.com; 4 Faculty of Technology and Metallurgy, University of Belgrade, 11000 Belgrade, Serbia; sseslija@tmf.bg.ac.rs

**Keywords:** pH-sensitive hydrogels, nanocellulose, controlled release

## Abstract

Hydrogels based on poly (methacrylic acid), carboxymethyl cellulose, and nanocellulose fibers were successfully synthesized, characterized, and tested as topical carriers for the controlled release of hydrophobic resveratrol and hydrophilic acetyl glucosamine, active substances used in skin protection. Carrier composition was confirmed by Fourier-transform infrared spectroscopy (FTIR). Scanning electron microscopy (SEM) revealed the pore size variations with alterations in the neutralization degree of methacrylic acid and changes in the pore-wall roughness caused by different mass fractions of nanocellulose. The neutralization degree of methacrylic acid had a substantial impact on the swelling behaviour, while only a slight change in swelling was caused by various contents of nanocellulose in hydrogels. Mechanical properties of the hydrogels accessed by compressive strength measurement at various percentages of strain were improved by the addition of nanocellulose. Hydrogels containing 0.5% nanocellulose achieved the highest compressive strength. The neutralization of methacrylic acid reduced the mechanical properties. Hydrogels with optimal properties showed outstanding potential in encapsulation, and controlled the simultaneous release of resveratrol and N-acetyl glucosamine. The different nature of the active compounds, however, affected the release kinetics and mechanism, as confirmed by the Korsmeyer–Peppas model.

## 1. Introduction

Hydrogels based on poly (methacrylic acid) (PMAA) have attracted considerable attention in biomedical research due to their high water absorption capacity, pH sensitivity, and excellent biocompatibility, making them suitable for applications such as drug delivery, tissue engineering, and medical implants [1]. One of the key parameters influencing their functional performance is the degree of neutralization of methacrylic acid (MAA), which directly affects hydrophilicity, mechanical properties, swelling behavior, and rheological characteristics [2].

In our previous studies, we have developed and characterized PMAA-based hydrogels that exhibit favorable properties such as high swelling capacity, environmental responsiveness, and the ability to provide controlled release of bioactive compounds. By adjusting polymerization parameters and crosslinker content, we have demonstrated that the structural and functional characteristics of these hydrogels can be finely tuned to match specific biomedical requirements [3,4]. Furthermore, the incorporation of nanocrystalline nanocellulose into the PMAA network has been shown to enhance mechanical strength and structural stability while simultaneously supporting sustained drug release profiles [5]. In addition, PMAA-based hydrogels have proved effective in delivering poorly soluble compounds—such as phenolic antioxidants and anticancer agents—underscoring their adaptability for controlled release systems [6].

Building upon these findings [3,5,6], the present study explores the effects of varying the degree of MAA neutralization and nanocellulose fibers (NC) content on the physicochemical and release properties of the resulting hydrogels. Nanocellulose, a biodegradable and biocompatible nanomaterial, has gained recognition for its ability to reinforce hydrogel networks, improve drug encapsulation efficiency, and enable sustained release [7,8]. However, its tendency to agglomerate presents a significant challenge for achieving uniform dispersion within polymer matrices. To address this, carboxymethyl cellulose (CMC) was introduced into the system. CMC improves nanocellulose dispersibility due to its viscoelastic nature and capacity to form homogeneous and stable hydrogel networks [9,10].

In this study, particular attention was given to the swelling behavior and controlled release performance of the PMAA-based hydrogels loaded with resveratrol and N-acetyl glucosamine—two therapeutically relevant compounds with complementary biomedical effects.

Resveratrol, a natural polyphenol, is widely recognized for its potent antioxidant properties, which enable it to scavenge free radicals and reduce oxidative stress, thereby protecting cells from damage. Beyond its antioxidant capacity, resveratrol exhibits significant anti-inflammatory effects by inhibiting the production of pro-inflammatory cytokines and enzymes, making it valuable in the treatment of various inflammatory skin conditions and other tissues [11]. Despite these beneficial effects, resveratrol’s clinical application is limited due to its poor aqueous solubility and rapid metabolism, which result in low bioavailability [12]. The incorporation of resveratrol into hydrogel matrices enhances its stability, protects it from premature degradation, and allows for controlled and sustained release. This approach is particularly advantageous for topical applications in dermatology and skin care, facilitating efficient delivery of its bioactive compounds directly to the affected tissues [13].

N-acetyl glucosamine, a derivative of glucose and a fundamental building block of glycosaminoglycans, has shown significant potential in tissue regeneration and wound healing. Its ability to stimulate extracellular matrix production and exert anti-inflammatory effects makes it a valuable candidate for incorporation into hydrogel-based delivery systems [14,15]. The controlled release of N-acetylglucosamine from PMAA-based hydrogels can further enhance its regenerative efficacy in applications such as cartilage repair and skin regeneration [16]. It is well known that resveratrol exhibits significant anti-inflammatory properties and supports skin barrier function by modulating cytokine expression and promoting keratinocyte [17]. N-acetyl glucosamine has also demonstrated therapeutic potential in dermatology by enhancing skin barrier repair, reducing inflammation, accelerating wound healing, and improving skin hydration through stimulation of hyaluronic acid [18].

Together with insights from the existing literature and our earlier results, this study aims to elucidate how the interplay between MAA neutralization, nanocellulose fiber reinforcement, and CMC-assisted dispersion affects hydrogel performance. Special attention is given to the structure–property relationships, where morphological, swelling, and mechanical characteristics are systematically correlated with formulation variables, offering a comprehensive understanding rarely addressed in similar studies. Importantly, the hydrogel system enables the simultaneous controlled release of two active substances- hydrophobic resveratrol and hydrophilic N-acetyl glucosamine—within a single matrix, overcoming a common challenge in topical delivery systems. This dual-delivery capability, combined with tunable mechanical and swelling behavior, positions the developed hydrogel as a promising multifunctional platform for future biomedical applications, including localized therapy, tissue repair, and regenerative medicine [19,20].

To comprehensively evaluate the structural and functional characteristics of the prepared hydrogels, a range of analytical techniques was employed. FTIR was used to confirm chemical interactions between hydrogel components, while SEM provided insights into the morphology and porosity of the hydrogel networks. Swelling experiments were conducted to assess water uptake behavior, followed by encapsulation and release studies of both resveratrol and N-acetyl glucosamine. For selected hydrogel formulations that demonstrated the most favorable properties in terms of structural stability and potential biomedical relevance, further advanced analyses were carried out. These included drug release experiments using a Franz diffusion cell to simulate transdermal delivery, as well as swelling and release kinetics modeling using the Korsmeyer–Peppas (K-P) model to determine the underlying transport mechanisms. Additionally, mechanical properties were evaluated through uniaxial compression tests, providing essential data on compressive strength relevant to implantable and load-bearing applications. By applying these characterization methods to the most promising hydrogel formulations, the study aims to establish a strong foundation for their further development and translation into biomedical practice.

## 2. Results and Discussion

### 2.1. FTIR and SEM Analysis of PMAA-Based Hydrogels

**Figure 1:**
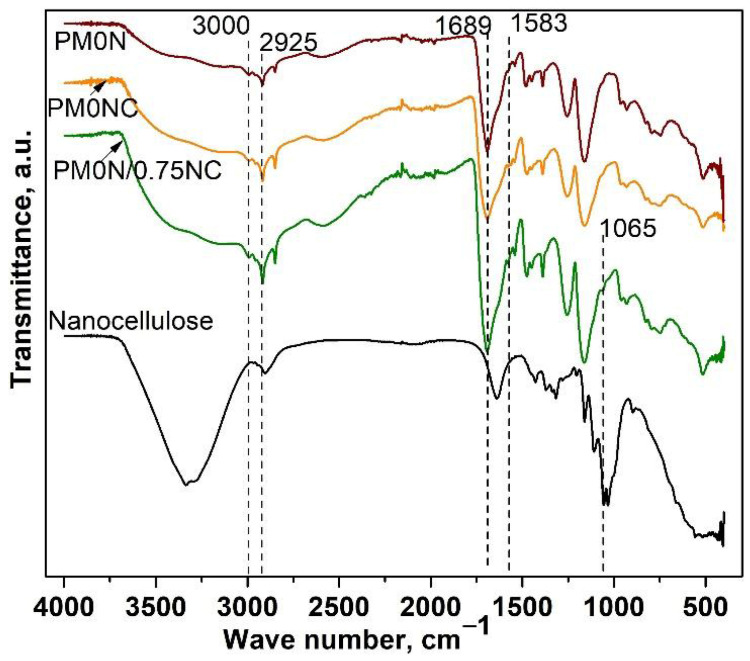
FTIR spectra of the PM0N, PM0N/0NC and PM0N/0.75NC hydrogels and nanocellulose.

FTIR analysis was employed to confirm the composition of the obtained PMAA-based samples. Recorded FTIR spectra of the PMAA-based samples are presented in Figure 1 FTIR spectra of PM0N, PM0N/0NC and PM0N/0.75NC samples revealed characteristic bands of PMAA: a wide band in a range of 3550 to 3000 cm^−1^ (OH stretching vibrations), at 3000 and 2925 cm^−1^ (stretching vibrations of methylene groups), and the band at 1689 cm^−1^ originating from C=O stretching vibrations (Figure 1) [21]. Characteristic bands of CMC were also present in the FTIR spectra of PM0N/0NC and PM0N/0.75NC samples (Figure 1): a small band at 1583 cm^−1^ (carboxyl group (COO^−^) stretching) [22]. Additionally, the CMC contribution to the FTIR spectrum of PM0N/0NC and PM0N/0.75NC reflected into the increase of the bands corresponding to the OH stretching vibrations (3550–3000 cm^−1^) and widening of the band of C=O stretching vibrations (around 1689 cm^−1^). Also, the presence of a characteristic band of NC at 1061 cm^−1^ (C-O stretching) in the FTIR spectrum of the PM0N/0.75NC sample was detected [23]. NC caused the additional rise and widening of the bands at 3550–3000 cm^−1^ and 1689 cm^−1^.

**Figure 2:**
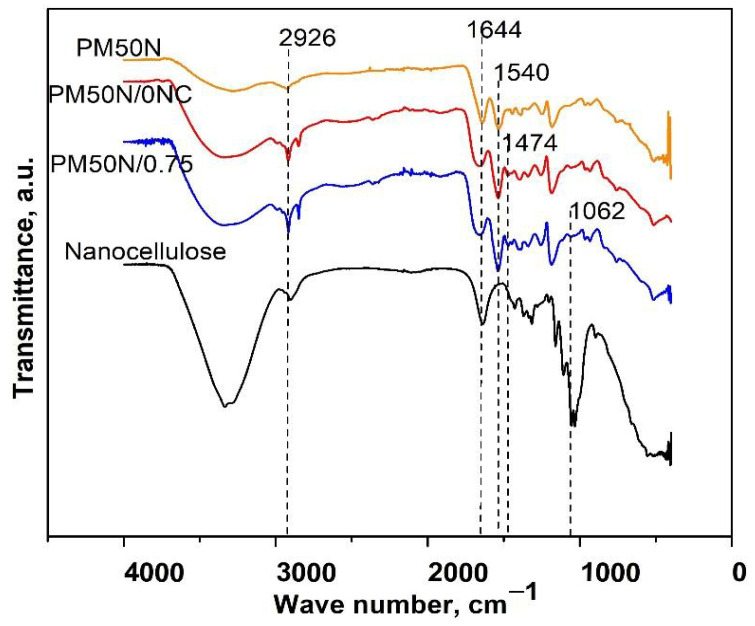
FTIR spectra of the PM50N, PM50N/0NC and PM50N/0.75NC hydrogels and nanocellulose.

Characteristic bands of PMAA were observed in the FTIR spectra of PM50N, PM50N/0NC, and PM50N/0.75NC: the wide band from 3550 to 3000 cm^−1^ originating from OH stretching vibrations, the band at 2926 cm^−1^ (stretching vibrations of methylene groups) and 1540 cm^−1^ (stretching vibrations of –COO^−^ groups) (Figure 2) [21]. FTIR analysis of PM50N/0NC and PM50N/0.75NC samples also revealed the presence of CMC by the increase and widening of the bands at 3550–3000 cm^−1^ and 1644 cm^−1^, respectively and appearance of a tiny band at 1474 cm^−1^ (C–H bending vibrations of methyl or methylene groups) [22]. The characteristic band of NC at 1065 cm^−1^ was present in the FTIR spectrum of PM50N/0.75NC (Figure 2) [23].

**Figure 3:**
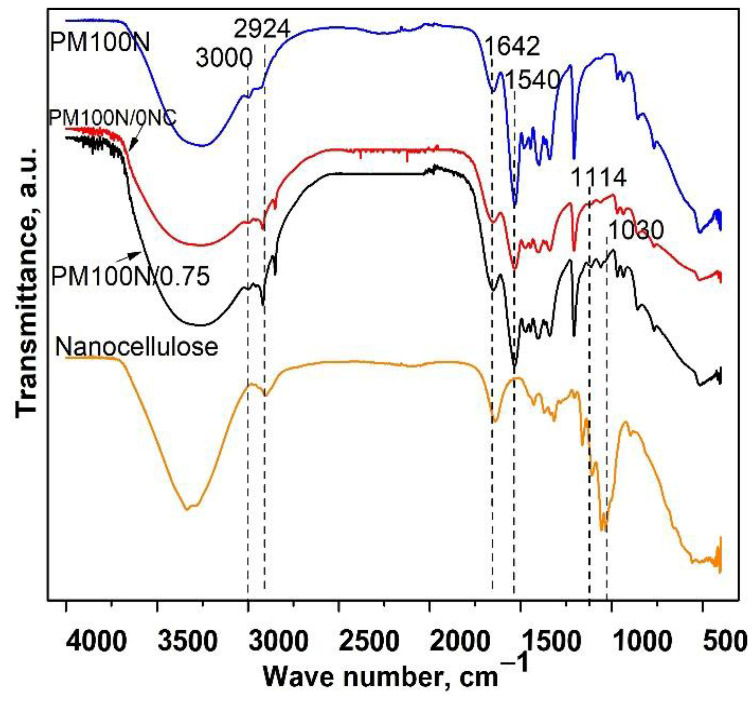
FTIR spectra of the PM100N, PM100N/0NC and PM100N/0.75NC hydrogels and nanocellulose.

FTIR spectra of the PM100N, PM100N/0NC, and PM100N/0.75NC samples showed the presence of characteristic bands of PMAA: the wide band from 3550 to 3000 cm^−1^ (OH groups), bands at 2926 cm^−1^ and 3000 cm^−1^ (methylene groups), and at 1540 cm^−1^ (–COO^−^ groups) (Figure 3) [21]. The band originating from C–O–C stretching vibrations of CMC at 1114 cm^−1^ was present in the FTIR spectra of PM100N/0NC and PM100N/0.75 samples [24]. FTIR spectrum of PM100N/0.75NC (Figure 3) revealed the existence of the characteristic bands of NC at 1030 cm^−1^ (-C-OH groups) [25]. The presence of CMC and NC also caused an increase in the intensity of the band at 3550–3000 cm^−1^ and contributed to the band at 1644 cm^−1^.

Increase in the intensity of the band at 1540 cm^−1^ and decline in the size of the band at 1644 cm^−1^ was observed, suggesting a predominance of the carboxylate group (–COO^−^) over carboxylic group (–COOH), resulting from neutralization of methacrylic acid (MAA) (Figure 1, Figure 2 and Figure 3) [26].

**Figure 4:**
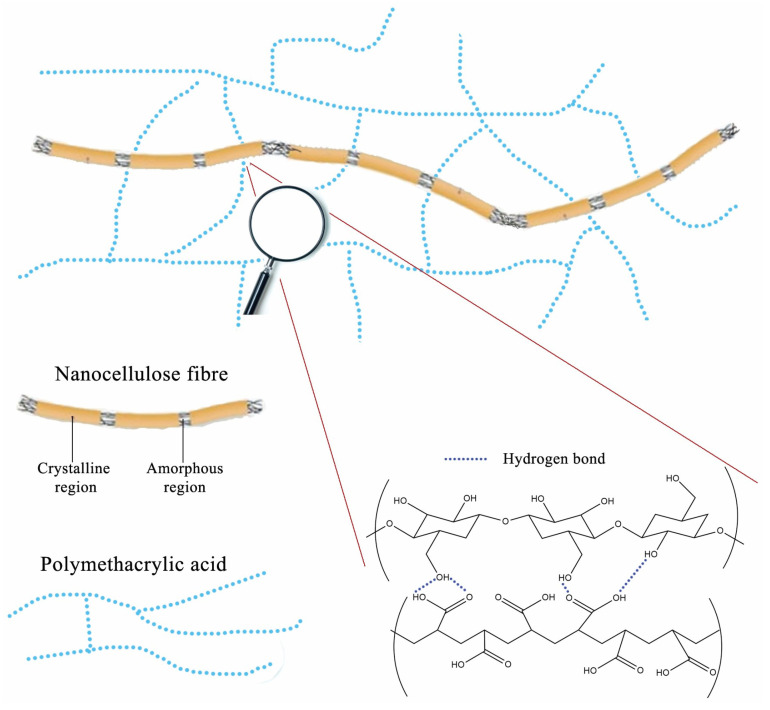
The schematic illustration of the proposed interactions between nanocellulose fibers and polymer network in PM0N/xNC hydrogel.

The schematic illustration of the proposed interactions between nanocellulose fibers and polymer network in PM0N/xNC hydrogel is presented in Figure 4.

**Figure 5:**
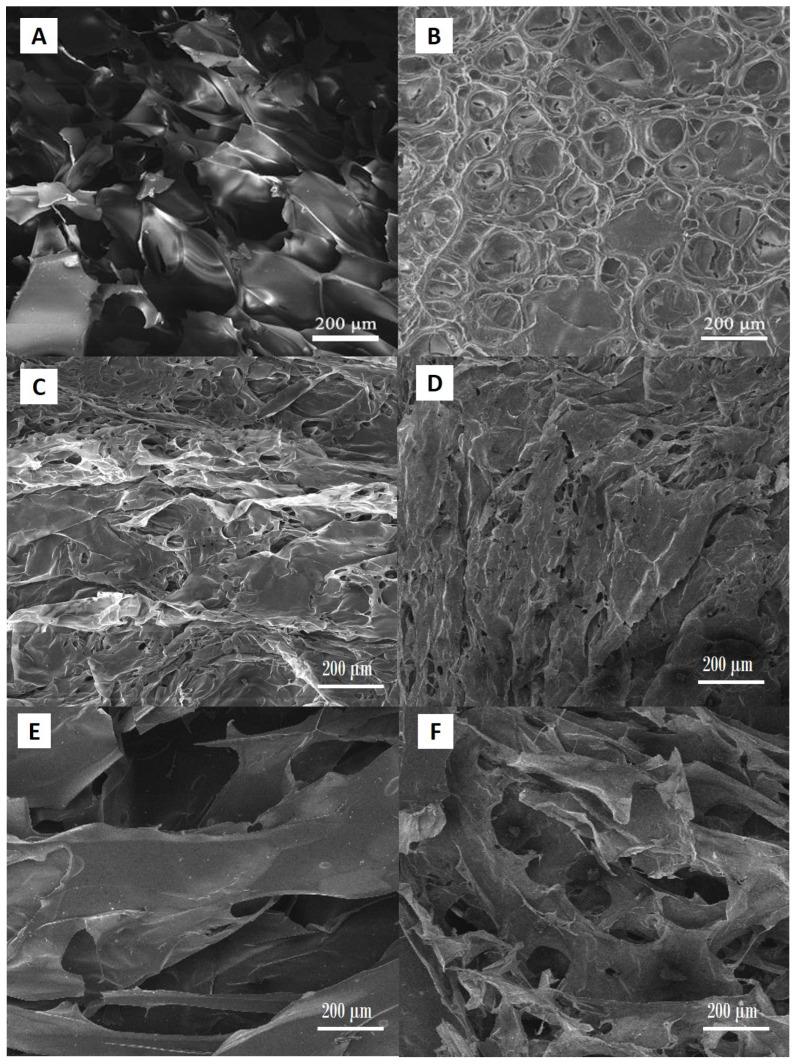
SEM micrographs of (**A**) PM0N/0NC, (**B**) PM0N/0.75NC, (**C**) PM50N/0NC, (**D**) PM50N/0.75NC, (**E**) PM100N/0NC and (**F**) PM100N/0.75NC samples.

The SEM analysis of the hydrogel samples showed regular porous structure characteristic for this type of materials (Figure 5A–F).

Scanning electron microscopy investigations demonstrated the effect of both NC content and the degree of neutralization of MAA on the microstructure of the hydrogels. Hydrogels with higher degrees of neutralization exhibited a substantially more open, interconnected porous network. This morphology is attributed to enhanced electrostatic repulsion between deprotonated carboxyl groups, leading to expanded polymer chain conformations during gelation and freeze-drying—a behavior consistent with observations in PAA/CNC and PMAA systems [27]. In contrast, samples with lower neutralization levels produced denser, less porous architectures. Reduced ionization limits swelling capacity, resulting in tighter polymer packing during cross-linking and drying [27]. Individual NC fibrils were not distinctly resolved in SEM micrographs captured at 200 µm scale; however, a clear trend emerged: with increasing NC mass fraction, amplified surface roughness of the pore walls was observed. This roughening effect likely stems from NC fibrils accumulating near the polymer matrix interface, producing a texturally richer morphology not visible at lower magnification but evident as increased microscale asperities under SEM.

These findings align with the broader literature asserting that filler content and ionic conditions critically govern pore topology in NC-reinforced polyelectrolyte hydrogels [28,29,30]. Notably, Yue et al. [29] have reported that NC incorporation in polyelectrolyte hydrogels promoted enhanced micro-roughness and stabilized pore walls, improving mechanical resilience. Their work has suggested that NC not only reinforced the polymer network but also contributed to heterogeneity at the pore surface, which may influence cell–material interactions in biomedical contexts. Our SEM results showed more irregular and textured pore surfaces as a result of the NC fraction increase, which is consistent with the structural stabilization and roughness enhancement discussed by Yue et al.

In summary, an increase in the degree of MAA neutralization leads to more open porosity, while boosting NC content enhances pore-wall roughness—even when individual fibrils are below the SEM resolution at 200 µm. This dual effect suggests a pathway to tailor hydrogel architecture by modulating both ionic strength and filler concentration, reflecting conclusions from recent studies [28,29,30].

### 2.2. Swelling of PMAA-Based Hydrogels

**Figure 6:**
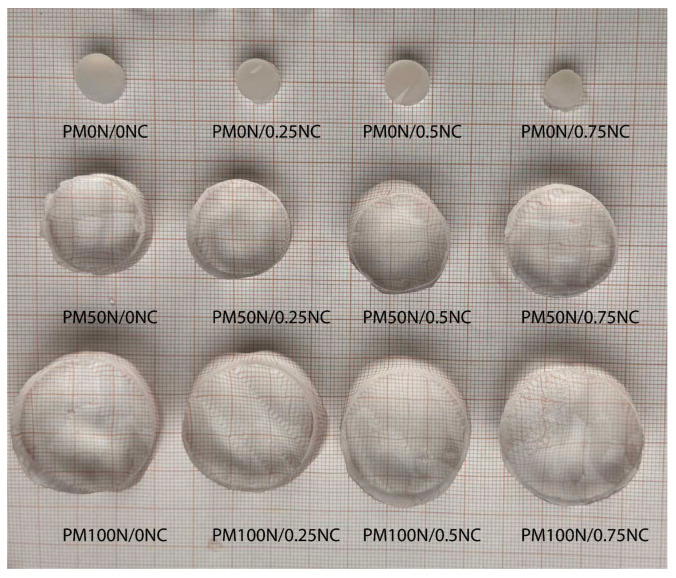
Images of PM0N/xNC, PM50N/xNC and PM100N/xNC, x = 0, 0.25, 0.5 and 0.75, hydrogels in the equilibrium state after swelling in distilled water at 25 °C.

One of the most important properties of hydrogels is their swelling behavior. This property is extremely important when using hydrogels for wound treatment, where there is a necessity to remove a certain amount of exudate from the wound while also providing conditions for effective healing, which involves maintaining appropriate moisture and preventing the dressing material from sticking to the wound. Additionally, if a hydrogel material is used as a carrier for an active substance, the swelling behavior also determines the amount of active substance that can be encapsulated in the hydrogel and the amount that will be released under application conditions. Since understanding of the swelling kinetics is crucial for evaluating the suitability of PMAA-based hydrogels as biomaterials for wound treatment, the swelling was investigated in detail. Figure 6 presents all three series of synthesized hydrogels in an equilibrium swollen state after swelling in distilled water at 25 °C.

**Figure 7:**
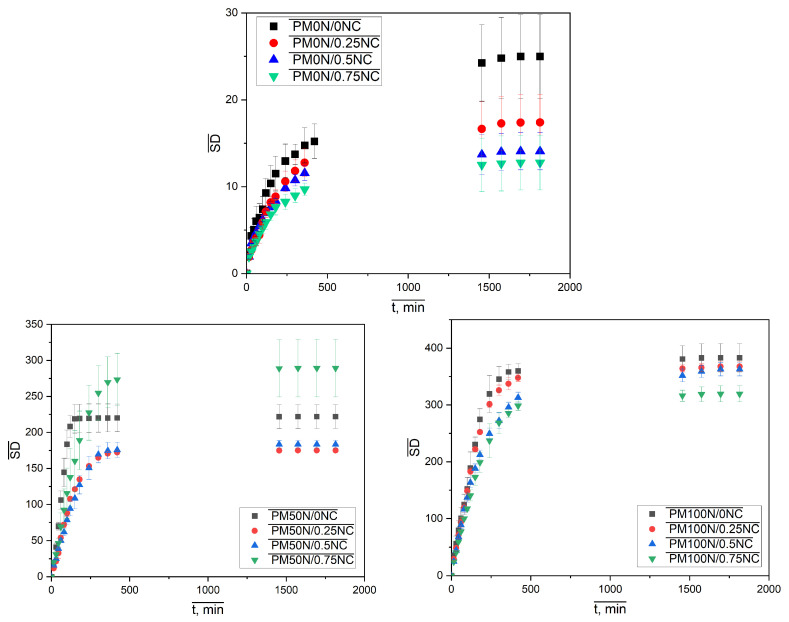
Isothermal swelling kinetic curves of PMAA-based hydrogels in distilled water at 25 °C.

As seen in Figure 6, the neutralization degree of MAA has a dominant influence on the amount of the water taken up, causing an evident increase in the sample diameter and height with increasing neutralization degree. The effect of variation in the mass fraction of NC fibers is not that obvious. To investigate this influence, as well as the swelling kinetics, the isothermal swelling curves of PMAA-based hydrogels are presented in Figure 7.

All kinetic curves have similar shapes, and all samples reached equilibrium within 24 h; what’s more, most of them reached 85–90% of their SDeq values within the first 8 h of swelling. The kinetic curves were transformed into dependencies of ln (SD/SDeq) on ln*t*, and swelling kinetic parameters *k* and *n* were determined according to the Peppas kinetic model. The obtained values are presented in Table 1.

Values of the parameter *k*, denoting the swelling rate constant, and the parameter *n*, indicating the mode of penetrant transport, were both more affected by the change in the neutralization degree than the NC mass fraction. The series of samples with 0N had the highest rate constants, whereas the parameter n was very close to 0.5, a value characteristic of Fickian diffusion. The neutralization of MAA led to an increase in the swelling rate constant and a change in the water penetration mechanism, since in both series, PM50N/xNC and PM100N/xNC, the values of the parameter n increased and were in most cases in a range of 0.5 < *n* < 1 indicating the non-Fickian diffusion. An increase in the NC mass fraction slightly affected the kinetic parameters. In the case of PM50N/0NC and PM50N/0.25NC samples, the *n* values were >1, indicating the super case II transport, where the swelling rate was controlled by relaxation of the polymer network. Similar findings have been reported in our previous work [31], where the swelling rate decreased with increasing neutralization up to a point, and then increased again, highlighting the complex influence of ionic interactions and polymer network dynamics.

**Figure 8:**
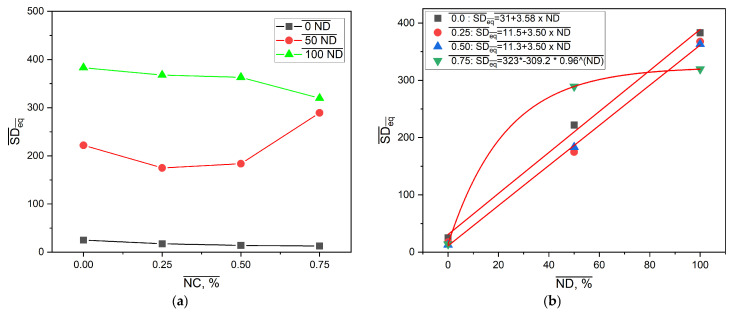
The effect of (**a**) neutralization degree of MAA and (**b**) NC mass fraction on SDeq in distilled water at 25 °C.

The effect of neutralization degree and NC mass fraction on the values of the equilibrium swelling degree is presented in Figure 8.

Results presented in Figure 8a are consistent with Figure 6. As expected, the SDeq values of the samples from the PM0N/xNC and PM100N/xNC series were substantially different. This finding is in accordance with the form of -COOH groups. In the neutralized PMAA network, repulsion between ionized -COO^−^ groups facilitates network expansion in contact with water molecules. On the other hand, PMAA without neutralization has mainly -COOH groups, H bonds inside the network (PMAA-PMAA and PMAA-NC) and a compact structure that swells very little. Furthermore, there is a difference in kinetics of polymerization between methacrylic acid and sodium methacrylate. It has been reported that total neutralization of MAA led to a lower gel phase i.e., under the same polymerization conditions, the PM100N network was less compact and swelled more than the PM0N network [31].

The same effect of increase in the NC mass fraction on SDeq was observed in these two series: the higher the NC content, the lower the SDeq. The NC mass fraction increase from 0 to 0.75% caused the decline in SDeq of 43.7% and 16.6% in the PM0N/xNC and PM100N/xNC series, respectively.

SDeq values of the PM50N/xNC series were in between those of the PM0N/xNC and PM100N/xNC series; however, a complex change in SDeq with NC mass fraction increase was observed. The incorporation of NC inside the PMAA network led to an SDeq decrease compared to the neat PMAA network, which can be attributed to the formation of interactions between PMAA carboxyl groups and NC hydroxyl groups, such as hydrogen bonds [6,7]. These physical crosslinks act as temporary junction points, which restrict chain mobility and limit network expansion and swelling However, further increase in NC mass fraction led to increase in SDeq. Polymer networks of the samples with the NC mass fraction of 0.50–0.75% were looser compared to PM50N/0.25, probably due to the larger number of NC-NC interactions relative to NC-PMAA interactions. At higher NC content (0.50–0.75%), the NC-NC interactions become more likely, either through hydrogen bonds between adjacent nanocellulose surfaces or via physical entanglement and aggregation. Such NC-NC interactions limits the availability of NC surface hydroxyl groups for interaction with PMAA chains, which reduce the density of effective polymer-filler crosslinking points. As a result, the polymer network becomes less efficiently reinforced and looser which allow greater swelling. The obtained results are consistent with the mechanical testing results (presented below these findings) and as expected, the samples with the lower SDeq exhibited better mechanical properties.

Figure 8b shows that correlations between SDeq and neutralization degree at constant NC mass fraction could be described with linear functions for 0–0.50% NC and exponential functions for 0.75% NC. The existence of these correlations allows for fine tailoring of the PMAA-based nanocomposites SDeq by making small changes in neutralization degree or NC amount in feed composition and thereby creating the drug carrier with desired behavior. The equilibrium swelling degree for sample PMXN/0.5NC increases by 1204.1% when the degree of neutralization is increased to 50%, and changes by 97.86% when the neutralization degree is increased to 100%.

### 2.3. Analysis of Mechanical Properties of PM0N, PM50N and PM/100N Samples

One of the most important properties of hydrogel dressings is their optimal mechanical properties, particularly their resistance to prolonged mechanical pressure. A hydrogel dressing resistant to external mechanical influences would not require frequent replacement, thereby minimizing repeated mechanical trauma to the wound during each dressing change, ultimately improving the wound healing process. Therefore, the mechanical properties of the PM0N/xNC, PM50N/xNC, and PM100N/xNC series of samples were analyzed, with particular focus on the influence of the addition of NC on the mechanical properties of the hydrogels. Compressive strength was evaluated at different percentages of strain after each of five consecutive compression cycles. The obtained compressive strength values for the PM0N/xNC, PM50N/xNC, and PM100N/xNC series of samples are presented in Table 2, Table 3 and Table 4. Compressive stress vs. cycle number plots for PM0N/xNC, PM50N/xNC, and PM100N/xNC hydrogels are presented in Figure S2, Supplementary Material.

The results presented in Table 2 show that the addition of NC fibers led to improvement in mechanical properties. With an increase in the NC mass fraction up to 0.5%, the compressive strength of the PM0N/xNC sample series increased in all compression cycles. However, with a further increase in NC content to 0.75%, a decrease in compressive strength of the PM0N/xNC series was observed, indicating a deterioration of mechanical integrity likely due to excessive filler content causing aggregation or matrix disruption. Furthermore, with the rising the percentage of strain (from 10 to 60%), the compressive strength values of the PM0N/xNC sample series also increased, suggesting that the samples offer greater resistance to the applied external compressive force at the higher value of the strain, likely due to densification and structural compaction mechanisms becoming more dominant at elevated deformations.

The analysis of LCS values reveals that all samples exhibit a certain degree of compressive strength degradation over five loading-unloading cycles, with the extent of loss increasing at higher strain levels. Notably, the sample with 0.5 wt% nanocellulose (PM0N/0.5NC) showed the highest initial strength but also significant strength loss at 50% and 60% strain, indicating reduced fatigue resistance under severe deformation. In contrast, the sample with 0.25 wt% NC demonstrated the most favorable balance between strength enhancement and stability across cycles.

Hydrogels based on 50% neutralized MAA exhibited inferior mechanical performance, with failure occurring at strain levels above 40%. This behavior is most likely a consequence of significantly higher swelling, which indicates that the polymer network is subjected to greater internal stress compared to non-neutralized counterparts, ultimately leading to structural failure under higher compressive deformation.

At 10% strain, all hydrogels exhibited relatively low compressive strength values and modest degradation across five cycles, with LCS values ranging from 3.5% to 9.3%. The most stable performance was observed for the sample with 0.25 wt% NC, indicating that a low nanocellulose content effectively enhances both strength and fatigue resistance under mild deformation.

At 20% strain, strength degradation became more pronounced. While PM50N/0.25NC showed the highest initial compressive strength, it also experienced a dramatic loss of 28.8%, pointing to poor cycle stability despite its stiffness. Conversely, PM50N/0.5NC showed better retention (LCS = 9.7%) with relatively high strength, suggesting a more favorable balance of rigidity and resilience at this strain level.

At 40% strain, all samples demonstrated significant structural breakdown. PM50N/0NC failed completely after the third cycle, and PM50N/0.75NC exhibited the highest LCS (85.3%), indicating severe fatigue-induced damage. Notably, PM50N/0.5NC retained moderate strength and showed lower degradation (LCS = 34.8%) compared to other NC-loaded samples, suggesting its superior performance under high-strain cyclic compression.

Hydrogels based on fully neutralized PMAA demonstrated the lowest deformation tolerance, with all samples failing beyond 10% compressive strain. This pronounced mechanical limitation is attributed to extensive swelling, which significantly increases internal osmotic pressure and network tension, rendering the polymer structure highly susceptible to failure even under mild compressive loading. Despite relatively high initial compressive strength values, the limited strain-bearing capacity reflects the fragility of the over-swollen network and its reduced ability to dissipate mechanical energy.

Since the PM50N/xNC and PM100N/xNC series were mechanically unstable at higher percentages of strain, further testing was accomplished with the PM0N/xNC series of samples that showed optimal mechanical properties and swelling behavior.

### 2.4. Controlled Release of Resveratrol and N-Acetyl Glycosamine from PMAA-Based Hydrogels

**Figure 9:**
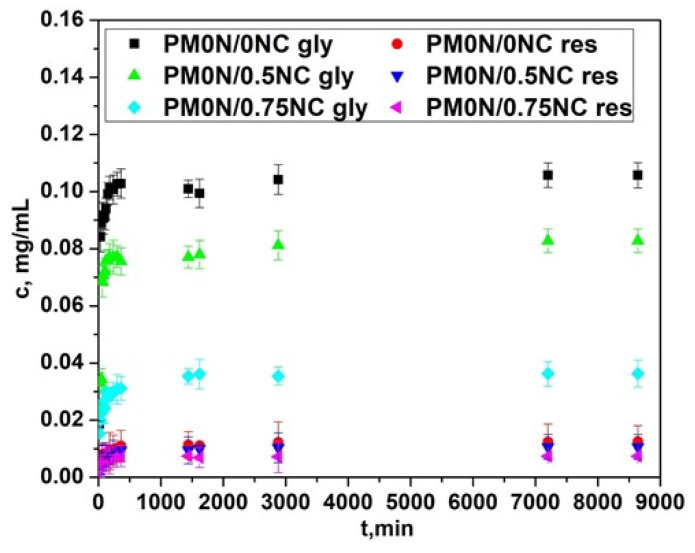
Controlled release of resveratrol and acetyl glucosamine from the PM0N/0NC, PM0N/0.5NC and PM0N/0.75NC hydrogels.

To improve wound healing, various types of active substances have been encapsulated in hydrogel dressings [32,33]. This approach can enhance bioavailability of the active substances and prolong their release time, thereby extending their therapeutic effect. Additionally, encapsulation of the active substance in hydrogels allows for controlled release kinetics, meaning that a specific therapeutic dose is released over a longer period of time in a controlled manner. Controlling the release kinetics of the active substance from the hydrogel dressing is a crucial factor for effective and safe wound treatment. Hence, to investigate the possibility of using PM0N/xNC hydrogels for controlled and prolonged release of active substances with wound-healing potential, resveratrol and N-acetyl glucosamine were encapsulated in the PM0N/xNC hydrogels. PM0N/xNC hydrogels were selected to evaluate the controlled release of active substances due to their optimal swelling behavior and mechanical properties. It is well known that resveratrol exhibits significant anti-inflammatory properties and supports skin barrier function by modulating cytokine expression and promoting keratinocyte [17]. N-acetyl glucosamine has also demonstrated therapeutic potential in dermatology by enhancing skin barrier repair, reducing inflammation, accelerating wound healing, and improving skin hydration through stimulation of hyaluronic acid [18]. The loading capacity (LC) was defined as the content of active substance (mg) per gram of hydrogel [34]. For N-acetyl glucosamine, the LC was approximately 0.20 ± 0.01 mg/g in all hydrogels. Similarly, the LC for resveratrol was 0.02 ± 0.002 mg/g, showing no dependence on the hydrogel type. The obtained release profiles of the active substances are presented in Figure 9.

The release profiles of both active compounds were affected by the presence of NC in the PM0N/xNC hydrogels. As seen in Figure 9, with increasing the mass fraction of NC, the concentrations of the released active compounds declined. As shown in Section 2.2 the NC content had minimal impact on the swelling behavior of the hydrogels. Thus, interactions between NC and the active compounds most probably played a key role in the release process. The slower release of the active substances observed with increase in NC content can be attributed to several factors. Nanocellulose is rich in hydroxyl groups, which can form hydrogen bonds both with the functional groups of the encapsulated active substances and with carboxylic acid groups present in the PMAA matrix [35]. These interactions can slow down diffusion of active substances from the carrier. When the PMAA chains is non-neutralized, –COOH groups are available to interact with the hydroxyl groups of NC, creating localized regions of tighter association within the hydrogel. These interactions lead to the formation of denser microdomains that further restrict the mobility of the encapsulated active substances and therefore, slow down their diffusion.

In addition to this effect, NC fibres can also act as physical obstacles within the hydrogel, forcing the molecules of active substances to navigate through more complex pathways, which slows down their overall diffusion. This steric hindrance, combined with the interactions of the active substances with NC-rich regions, causes some of the molecules to become temporarily entrapped in these domains, slowing their release. As a result, a fraction of the active compounds is retained longer and released more slowly, until the noncovalent interactions are gradually overcome.

Based on the above-presented results, the hydrogel with the optimal properties (the adequate release profiles of the active compounds and the most promising mechanical properties and swelling behavior), PM0N/0.5NC was further used to evaluate the active compound diffusion from hydrogel in vitro in Franz cell.

### 2.5. Franz Cell Experiments

Release of the active substances is not only dependent on the swelling behavior of the hydrogels but also on the properties of the specific active substances involved [34,36,37]. In this study, we used two active substances: one hydrophilic (N-acetyl glucosamine) and one hydrophobic (resveratrol). The release of both active substances from the hydrogel was monitored over a 6-day period using a Franz diffusion cell equipped with an acetate-cellulose membrane. Acetate-cellulose membranes are widely employed as a simple and reproducible model for controlled drug release in topical formulations. The properties of these membranes make them ideal for evaluating drug diffusion from hydrogels in vitro, offering a practical compromise between complex skin models and simpler solutions.

**Figure 10:**
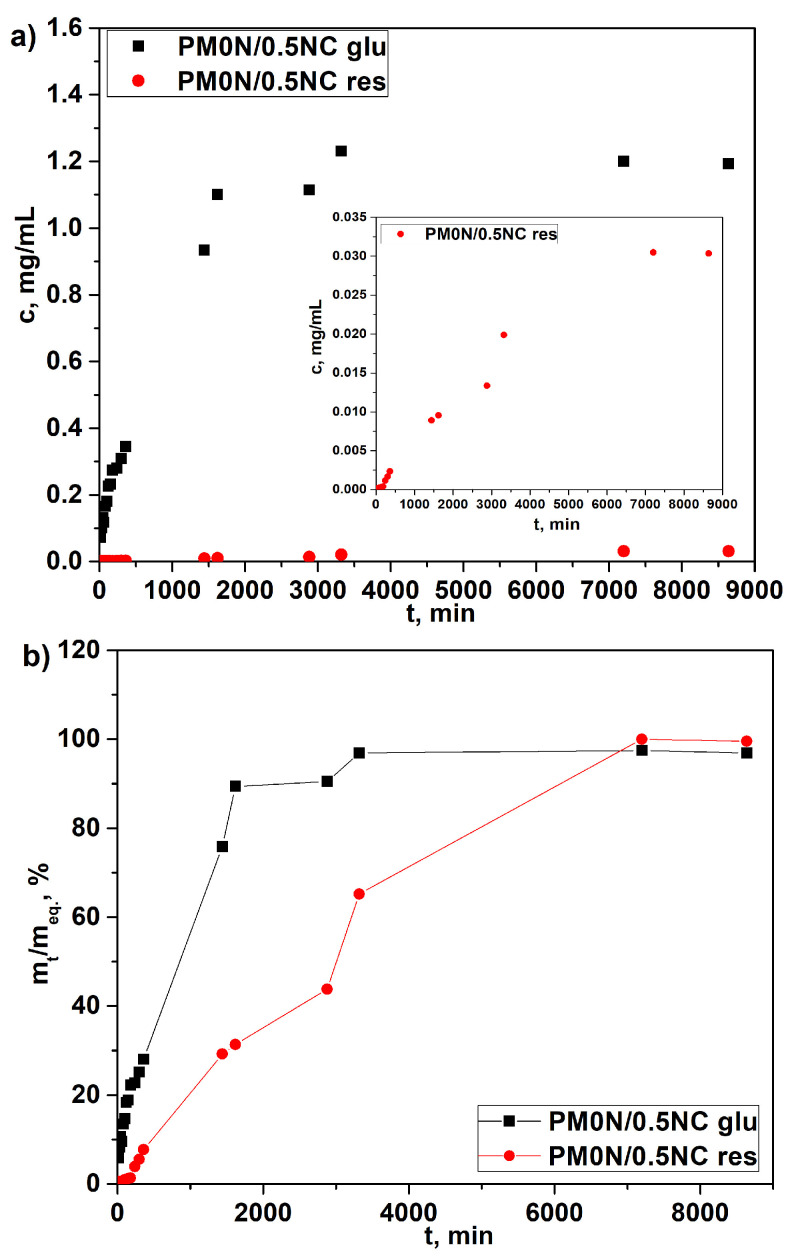
(**a**) The release profiles of acetyl glucosamine and resveratrol from the PM0N/0.5NC hydrogel and (**b**) cumulative release of acetyl glucosamine and resveratrol from the PM0N/0.5NC hydrogel.

Release profiles, depicted in Figure 10a, show that the active compounds release occurred in a highly controlled and sustained manner, which is a critical requirement for the intended application of the hydrogel. As shown in Figure 10a, the concentration of released N-acetyl glucosamine reached its maximum after 48 h. The concentrations of released resveratrol were much lower due to its low solubility in water, which hindered diffusion. To compare the release rates of two active substances, the release data are presented in Figure 10b as cumulative release vs. time. It is evident that N-acetyl glucosamine release reached a plateau much more quickly than resveratrol. These findings demonstrate the ability of the PM0N/0.5NC hydrogel to gradually release both hydrophilic and hydrophobic compounds, making it suitable for prolonged release in practical applications.

**Figure 11:**
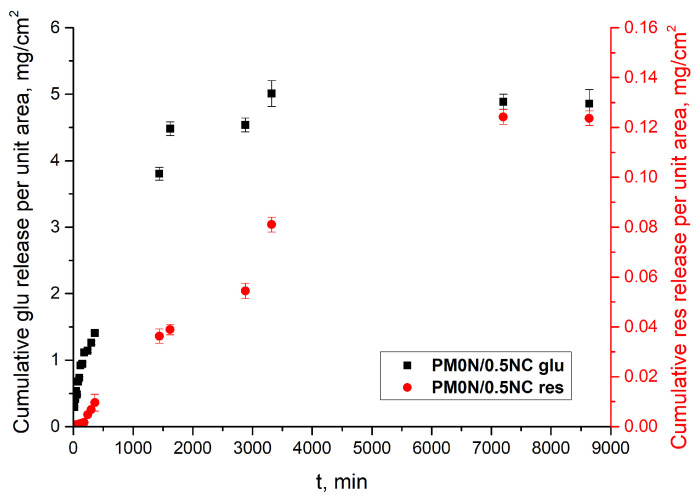
Flux profiles of N-acetyl glucosamine and resveratrol.

Figure 11 illustrates the flux profiles of N-acetyl glucosamine and resveratrol observed in this study. The calculated steady-state flux for N-acetyl glucosamine was 1.52 ± 0.9 mg cm^−2^ h^−1^, while for resveratrol, it was significantly lower 0.01 ± 0.001 mg cm^−2^ h^−1^. The flux of active substance across an acetyl cellulose membrane in a Franz diffusion cell is influenced by both the physicochemical properties of the active substances and the characteristics of the membrane [38,39]. Cellulose-based membranes generally exhibit lower drug flux compared to synthetic microfiltration membranes, primarily due to their higher resistance to diffusion.

Drug release from PM0N/0.5NC hydrogels was analyzed using the Korsmayer–Peppas (K–P) model. Release kinetics parameters, *n* and *k*, were determined from the intersects and slopes of the corresponding dependencies ln*α* vs. ln*t* (Figure S3, Supplementary Material) and are presented in Table 5.

The obtained results were aligned with previous findings from the release profiles and confirmed the significant effect of the nature of the active substance on its release from the carrier. Namely, the release rate, i.e., parameter *k*, was notably higher for N-acetyl glucosamine, which is a hydrophilic compound. Parameter *n* revealed a difference in the release mechanism; while in the case of N-acetyl glucosamine it was governed by Fickian diffusion (*n* ≈ 0.5), in the case of resveratrol, the Case II transport occurred (*n* ≥ 1), indicating the dominant effect of polymer relaxation on release kinetics. These distinct release behaviors can be attributed to differences in the molecular structure, solubility, and interactions of the drugs with the hydrogel network during the swelling process. Specifically, N-acetyl glucosamine is a small, highly water-soluble molecule with limited affinity for the hydrophobic regions of the polymer. Consequently, it dissolves quickly and diffuses through the aqueous channels formed during the initial swelling phase. This results in Fickian diffusion, where the release rate is primarily controlled by the concentration gradient and diffusion through water-filled pores. In contrast, resveratrol is a hydrophobic molecule with low water solubility and a stronger affinity for the less polar regions of the polymer matrix. As water enters the hydrogel and the network begins to swell, polymer chain relaxation enables the gradual release of resveratrol, which was initially entrapped or associated with hydrophobic domains. This corresponds to Case II transport, where the release is controlled by the rate of polymer relaxation rather than diffusion. The so-called range of applicability of the K-P model (*α*) was acceptable for both active substances, as it was higher than 60%, as well as the correlation factor R^2^, which had high values in both cases. These two parameters justify the use of the K-P model for the description and prediction of the release of both active substances from the investigated PM0N/0.5NC hydrogel.

## 3. Conclusions

In conclusion, this study provides a comprehensive analysis of how MAA neutralization, nanocellulose fiber reinforcement, and CMC-assisted dispersion collectively influence the structural, swelling, and mechanical properties of poly (methacrylic acid)-based hydrogels. By systematically correlating formulation parameters with performance characteristics, we establish detailed structure–property relationships that offer deeper insight compared to previous studies.

As revealed by the SEM analysis and swelling studies, the neutralization degree had a substantial impact on the size of pores and swelling degree of hydrogels, whereas the nanocellulose mass fraction affected the morphology of the pores edge. However, higher neutralization degrees resulted in hydrogels with weak polymer networks. A key outcome is the demonstration of the simultaneous controlled release of both hydrophobic resveratrol and hydrophilic N-acetyl glucosamine from a single hydrogel matrix—an approach that addresses a well-known limitation in topical drug delivery systems. It was also shown that the developed hydrogels served as effective carriers for both actives; however, their distinct physicochemical properties influenced the release kinetics and underlying mechanisms. The ability to fine-tune the hydrogel’s mechanical strength and swelling behavior further enhances its versatility. Overall, the developed hydrogel platform shows strong potential for multifunctional biomedical applications, including localized therapy, skin regeneration, and tissue repair. Future perspectives for carrier development will include fine-tuning of their properties, beginning with the further optimization of the degree of neutralization and associated mechanical characteristics.

## 4. Materials and Methods

### 4.1. Materials

Methacrylic acid (MAA) (99.5%) was purchased from Merck (Darmstadt, Germany). The crosslinker N, N′-methylenebisacrylamide (MBA) (p.a.) was supplied from Aldrich Chemical Co. (Milwaukee, WI, USA). Carboxymethyl cellulose—9M31F (CMC) (99.5%) was purchased from Ashland (Wilmington, DE, USA). Celova^®^ M250R-G from Weidmann Fiber (Rapperswil Switzerland) was used as a source of cellulose nanofibers (NC). Resveratrol was purchased from ChromaDex (Irvine, CA, USA). N-acetyl glucosamine was supplied from Avena Lab, Farmadria d.o.o. (Vršac, Serbia) Initiator, VA-044 (2,2′-Azobis [2-(2-imidazolin-2-yl)propane]dihydrochloride) (p.a.) was procured from Wako Pure Chemical Industries, Ltd., Osaka, Japan. All chemicals were of analytical grade and used as received.

### 4.2. Synthesis of PMAA-Based Hydrogels

PMAA-based hydrogels were prepared as follows. The total volume of the reaction mixture for each sample was 20 mL. Into the appropriate volume of distilled water (Table 6), 4 mL of methacrylic acid was added under constant stirring. For the samples with neutralized methacrylic acid, the corresponding amount of sodium hydroxide was added to the reaction mixture and stirred until sodium hydroxide was completely dissolved (Table 6). After the crosslinker MBA (0.4 mol% with respect to MAA) was added and dissolved, the corresponding mass of the aqueous nanocellulose solution (Table 6) was added under continuous stirring. The mass fraction (wt.%) of NC was calculated based on the dry matter content of the final mixture. Then, 2.5 g of a 4% (*w*/*v*) aqueous solution of carboxymethyl cellulose (CMC-SM31F) was added, and the reaction mixture was sonicated in an ultrasonic bath at room temperature until achieving a homogeneous state. Subsequently, 1.35 mL of a 1% solution of the initiator VA-044 was added, and stirring continued for another 5 min. After that, the reaction mixture was poured into glass molds (glass plates 17 × 17 cm, separated by 2 mm thick PVC strips) and left in an oven at 60 °C for 5 h. Subsequently, the samples were cut into disk shapes with a diameter of 7 mm, dried at room temperature, and used for further analysis.

The prepared hydrogels were labelled as PMxN/xNC, where xN denotes the degree of the methacrylic acid neutralizationand xNC the mass fraction of nanocellulose fibers. The hydrogel based only on non-neutralized poly (methacrylic acid) was marked with PM0N, whereas the sample with non-neutralized poly (methacrylic acid) and CMC but without nanocellulose was referred to as PM0N/0NC. Similarly, the hydrogel composed of 50% neutralized poly (methacrylic acid) was designated as PM50N, while the corresponding sample containing CMC but no nanocellulose was labelled as PM50N/0NC. When concerning the hydrogels with 100% neutralized methacrylic acid, the sample containing only neutralized methacrylic acid was denoted as PM100N, whereas the one with CMC and without nanocellulose was labelled as PM100N/0NC.

### 4.3. Gel Fraction Measurement

The efficiency of hydrogel network formation can be quantitatively evaluated using the gel fraction measurement [40]. The gel fraction percentage of the samples was then calculated using the following formula:

Gel fraction (%) = (W_d_/W_0_) × 100 (1)

where W_0_ refers to the initial weight of the dried sample, while W_d_ represents the weight of the dried insoluble portion of the sample after it has been extracted with water.

Determined values of gel fractions for each sample are presented in Table S2 in Supplementary material.

### 4.4. Methods

The Fourier Transform Infrared (FTIR) spectra of disk-shaped PMAA-based xerogels were recorded in transmittance mode in the wavelength range of 4000 to 500 cm^−1^ with a resolution of 4 cm^−1^ using a Nicolet™ iS10 FTIR Spectrometer Thermo Fisher Scientific, Madison, WI, USA.

Prior to SEM analysis, the PMAA-based samples were equilibrated in distilled water at room temperature. After reaching the swelling equilibrium, the samples were freeze-dried. Subsequently, they were fractured in half and coated with a gold–palladium alloy using a POLARON SC502 sputter coater Quorum Technologies, Laughton, East Sussex, UK. The resulting cross-sections were examined using a Tescan MIRA 3 XMU Field Emission Scanning Electron Microscope, Tescan, Brno, Czech Republic operated at an accelerating voltage of 20 kV.

Cyclic compression tests were performed to determine the mechanical properties of the disc-shaped PMAA-based hydrogels. In order to apply these tests, the PMAA-based samples were first swollen to equilibrium in distilled water at room temperature. After withdrawing the samples, the excess of water from their surfaces was gently removed by filter paper. The cyclic compression tests were further applied by using a Shimadzu Autograph AGS-X, Kyoto, Japan (1 kN) testing machine at a constant strain rate of 10 mm/min and room temperature. Cyclic compression tests were conducted in 5 consecutive cycles up to 10%, 20%, 40%, 50%, and 60% strain, at a strain rate of 10 mm/min, without any pause between successive cycles. The results of the cyclic compression test are given as an average value of compressive strength (σC) from three independent measurements for each cycle. Loss of compressive strength (LCS) after 5 cycles was calculated according to the following equation:

LCS = (σC1 − σC5)/σC1 × 100(2)

where σC1 is compressive strength after the first cycle and σC5 is compressive strength after the fifth cycle.

### 4.5. Swelling of PM0N, PM50N and PM100N Hydrogels

The equilibrium swelling degree of the PMAA-based hydrogels was determined gravimetrically using the tea bag method [41]. Dry discs (m_0_) were immersed in excess distilled water at 25 °C, removed at determined time intervals and weighed (m_t_). Swelling degree (SD) was calculated from the expression:

SD = (m_t_ − m_0_)/m_0_ (3)

The equilibrium SD (SD_eq_) was obtained when the constant mass (m_eq_) was accomplished. All measurements were done in triplicate and presented as their mean values. Derived swelling data were analyzed by the well-known Peppas kinetic model SD/SD_eq_ = kt^n^ from which linear form ln (SD/SD_eq_) = ln*k* + *n* ln*t*, parameters *k* and *n* were determined.

### 4.6. Encapsulation and Release of Resveratrol and N-Acetyl Glucosamine from Hydrogels

Encapsulation of the active substances was performed as follows: 0.04 g of resveratrol and 0.30 g of N-acetyl glucosamine were dissolved in 30 mL of 40% ethanol solution The samples were immersed in this solution and left to swell to equilibrium while protected from light. Subsequently, the samples were removed, excess liquid was blotted off with paper towels, and the samples were placed in distilled water to monitor release of the encapsulated active substances. The release studies were conducted in a batch system under gentle shaking, using distilled water as a release medium. Solutions of active substances were collected from the batch system at predetermined time intervals. The release of N-acetyl glucosamine and resveratrol was determined by measuring absorbance at 196 nm and 306 nm, respectively, up to the point at which the maximum release was achieved and stabilized. Based on the measured concentrations, release curves were constructed. The release of the active substances was monitored by using a UV-1800 spectrophotometer (Shimadzu, Kyoto, Japan).

### 4.7. Franz Cell

The in vitro release study was conducted using a Franz diffusion cell (courtesy of PermeGear, Inc., Hellertown, PA, USA), consisting of two compartments (donor and acceptor) separated by an acetate-cellulose membrane with a pore size of 0.2 μm [32,33]. Prior to the experiment, the membrane was soaked in the acceptor medium (distilled water) for 0.5 h. Approximately 1.5 g of the PM0N/0.5NC gel sample, with a thickness of approximately 3 mm, was placed in the donor compartment, while the receptor compartment was filled with distilled water and maintained at 25 °C. Continuous mixing at 360 rpm was provided by magnetic stirring [33]. Samples were collected from the receptor compartment at predefined time intervals until maximum release was achieved. The release of active compounds was monitored spectrophotometrically using a UV-1800 spectrophotometer (Shimadzu, Kyoto, Japan).

Drug release from PMAA-based hydrogels was analyzed using the Korsmeyer–Peppas (K–P) model:

(4) $$\alpha =k\times t^{n}$$

where, α = m_t_/m_eq_ refers to the fraction of drug released at time *t*, the constant *k* determines the rate of drug release and the exponent *n* characterizes the mechanism of release—whether it is primarily diffusion, polymer-chain relaxation, or a combination thereof.

For a planar carrier, the interpretation of *n* is as follows:

- *n* ≤ 0.5—drug release is dominated by Fickian diffusion.
- 0.5 < *n* < 1.0—anomalous (non-Fickian) transport, indicating both diffusion and relaxation contribution.
- *n* ≥ 1.0—release is governed by polymer relaxation, known as Case II transport
- *n* > 1 is termed super Case II transport, reflecting accelerated polymer relaxation effects.

The cumulative amount of active substances permeated per unit diffusion area was plotted against time, and the steady-state flux (J, mg/cm^2^·h) was determined from the linear portion of the permeation profile. Flux was calculated using the following equation:

J = dQ/(A · dt)(5)

where Q is the amount of active substance that permeated through the membrane of surface area A during the time interval *t*.

## Figures and Tables

**Table 1:** Swelling kinetic parameters.

NC	0N		50N		100N	
	*k*/min^−1^	*n*	*k*/min^−1^	*n*	*k*/min^−1^	*n*
0	0.023	0.56	0.0035	1.18	0.0092	0.83
0.25	0.020	0.60	0.0039	1.04	0.0070	0.88
0.50	0.036	0.54	0.0089	0.83	0.0080	0.82
0.75	0.033	0.54	0.0055	0.91	0.0081	0.83

**Table 2:** Compressive strength of the PM0N/xNC series of samples at different percentages of strain.

**Sample**	**(Compressive Strength at 10% Strain) kPa**					* **LCS, %**
	**First Cycle**	**Second Cycle**	**Third Cycle**	**Fourth Cycle**	**Fifth Cycle**	
PM0N/0NC	1.586	1.522	1.500	1.491	1.465	7.63
PM0N/0.25NC	2.085	2.057	2.099	2.055	2.033	2.49
PM0N/0.5NC	6.093	5.952	5.865	5.011	5.761	5.45
PM0N/0.75NC	5.232	5.202	5.810	5.071	5.056	3.36
**Sample**	**(Compressive Strength at 20% Strain) kPa**					* **LCS, %**
	**First Cycle**	**Second Cycle**	**Third Cycle**	**Fourth Cycle**	**Fifth Cycle**	
PM0N/0NC	5.821	5.714	5.701	5.603	5.626	3.35
PM0N/0.25NC	6.333	6.249	6.132	6.093	6.038	4.66
PM0N/0.5NC	17.845	17.191	16.996	16.787	16.736	6.21
PM0N/0.75NC	14.363	14.101	13.791	13.662	13.553	5.64
**Sample**	**(Compressive Strength at 40% Strain) kPa**					* **LCS, %**
	**First Cycle**	**Second Cycle**	**Third Cycle**	**Fourth Cycle**	**Fifth Cycle**	
PM0N/0NC	21.912	21.377	21.172	21.014	20.928	4.49
PM0N/0.25NC	31.216	29.645	29.431	29.233	29.137	6.66
PM0N/0.5NC	138.02	131.15	126.96	124.57	122.03	11.59
PM0N/0.75NC	89.014	84.371	82.015	80.325	79.445	10.75
**Sample**	**(Compressive Strength at 50% Strain) kPa**					* **LCS, %**
	**First Cycle**	**Second Cycle**	**Third Cycle**	**Fourth Cycle**	**Fifth Cycle**	
PM0N/0NC	49.458	47.178	46.075	45.834	45.763	7.47
PM0N/0.25NC	71.821	68.352	67.867	67.376	67.281	6.32
PM0N/0.5NC	430.06	380.99	354.23	316.09	286.05	33.49
PM0N/0.75NC	246.21	235.05	228.91	224.14	203.22	17.46
**Sample**	**(Compressive Strength at 60% Strain) kPa**					* **LCS, %**
	**First Cycle**	**Second Cycle**	**Third Cycle**	**Fourth Cycle**	**Fifth Cycle**	
PM0N/0NC	91.822	73.365	72.346	69.328	67.757	26.21
PM0N/0.25NC	196.05	136.65	116.44	109.30	107.24	45.30
PM0N/0.5NC	1581.7	1239.5	1151.5	1100.5	1043.0	34.06
PM0N/0.75NC	692.34	506.64	481.48	440.49	412.12	40.47

*LCS—Loss of compressive strength.

**Table 3:** Compressive strength of the PM50N/xNC series of samples at different percentages of strain (the mechanical properties of the PM50N/xNC series of samples at 40%, 50%, and 60% strain could not be analyzed due to samples failure during the first compression cycle).

**Sample**	**(Compressive Strength at 10% Strain) kPa**					* **LCS, %**
	**First Cycle**	**Second Cycle**	**Third Cycle**	**Fourth Cycle**	**Fifth Cycle**	
PM50N/0NC	0.66	0.64	0.63	0.61	0.60	9.09
PM50N/0.25NC	2.54	2.52	2.50	2.48	2.45	3.54
PM50N/0.5NC	2.25	2.20	2.17	2.17	2.11	6.22
PM50N/0.75NC	1.40	1.35	1.31	1.29	1.27	9.29
**Sample**	**(Compressive Strength at 20% Strain) kPa**					* **LCS, %**
	**First Cycle**	**Second Cycle**	**Third Cycle**	**Fourth Cycle**	**Fifth Cycle**	
PM50N/0NC	1.73	1.69	1.61	1.58	1.55	10.40
PM50N/0.25NC	6.42	6.18	5.95	5.71	4.57	28.82
PM50N/0.5NC	5.04	4.90	4.76	4.69	4.55	9.72
PM50N/0.75NC	3.23	3.17	3.10	3.05	2.99	7.43
**Sample**	**(Compressive Strength at 40% Strain) kPa**					* **LCS, %**
	**First Cycle**	**Second Cycle**	**Third Cycle**	**Fourth Cycle**	**Fifth Cycle**	
PM50N/0NC	3.15	2.15	1.53	Failed	Failed	/
PM50N/0.25NC	7.93	2.98	2.81	2.06	1.89	76.17
PM50N/0.5NC	9.22	8.18	7.79	5.92	6.01	34.82
PM50N/0.75NC	5.73	3.06	0.91	0.87	0.84	85.34

*LCS—Loss of compressive strength.

**Table 4:** Compressive strength of the PM100N/xNC series of samples at different percentages of strain (the mechanical properties of the PM100N/xNC series of samples at 40%, 50%, and 60% strain could not be analyzed due to samples failure during the first compression cycle).

Sample	(Compressive Strength at 10% Strain) kPa					*LCS, %
	First Cycle	Second Cycle	Third Cycle	Fourth Cycle	Fifth Cycle	
PM100N/0NC	16.76	16.43	16.13	15.96	15.74	6.09
PM100N/0.25NC	15.49	15.23	14.92	14.70	14.51	6.33
PM100N/0.5NC	13.57	13.36	13.17	12.99	12.87	5.16
PM100N/0.75NC	15.98	15.67	15.43	15.25	15.10	5.51

*LCS—Loss of compressive strength.

**Table 5:** Release kinetics parameters of K-P model.

Sample	*k*, min^−1^	*n*	*R* ^2^	α, %
PM0N/0.5NC glu	1.2 × 10^−2^	0.52	0.989	76
PM0N/0.5NC res	6.7 × 10^−5^	1.11	0.986	65

**Table 6:** Feed composition.

Samples	MAA, mL	NaOH, g	NC, wt%	4% Aqueous Solution of CMC, g	H_2_O, mL
PM0N	4	0	0	0	11.77
PM0N/0NC	4	0	0	2.5	9.73
PM0N/0.25NC	4	0	0.25	2.5	8.08
PM0N/0.5NC	4	0	0.5	2.5	6.43
PM0N/0.75NC	4	0	0.75	2.5	4.78
PM50N	4	0.9295	0	0	11.28
PM50N/0NC	4	0.9295	0	2.5	8.78
PM50N/0.25NC	4	0.9295	0.25	2.5	7.13
PM50N/0.5NC	4	0.9295	0.5	2.5	5.48
PM50N/0.75NC	4	0.9295	0.75	2.5	3.83
PM100N	4	1.859	0	0	10.33
PM100N/0NC	4	1.859	0	2.5	7.83
PM100N/0.25NC	4	1.859	0.25	2.5	6.18
PM100N/0.5NC	4	1.859	0.5	2.5	4.53
PM100N/0.75NC	4	1.859	0.75	2.5	2.88

## Data Availability

The original contributions presented in this study are included in the article/Supplementary Materials. Further inquiries can be directed to the corresponding author.

## Supplementary Materials

The following supporting information can be downloaded at: https://www.mdpi.com/article/10.3390/gels11100838/s1, Figure S1. Images of PM0N/xNC, PM50N/xNC and PM100N/xNC, x = 0, 0.25, 0.5 and 0.75, hydrogels in the equilibrium state after swelling in distilled water at 25°C; Figure S2. Compressive stress vs. cycle number for: (a) PM0N/xNC, (b) PM50N/xNC, and (c) PM100N/xNC; Figure S3. Determination of the release kinetics parameters from K-P model; Figure S4. Swelling curves in pH5.5; Table S1. Comparison on swelling degree, compressive strength, and release profiles in recent key studies [42,43,44,45,46,47]; Table S2. Gel fraction; Table S3. Peppas kinetic parameters for the swelling in pH5.5.
