# Supplementary material for: Sustainable Poly (Methacrylic Acid)/Nanocellulose Hydrogel for Controlled Simultaneous Release of Active Substances for Skin Protection
Source: Gels. 2025 Oct 18;11(10):838. doi: 10.3390/gels11100838 (PMC12563752; doi:10.3390/gels11100838)
Supplement: Supplementary file 1 [file gels-11-00838-s001.zip › gels-3893293-supplementary.pdf]

Supplementary data

Sustainable Poly (Methacrylic Acid)/Nanocellulose Hydrogel for Controlled Simultaneous Release of Active Substances for Skin Protection

Katarina Antic [1,*], Maja Markovic [1], Vesna Panic [1], Pavle Spasojevic [1,2], Bojana Balanc [1], Milica Spasojevic Savkovic [3] and Sanja Savic [4]

[1] Innovation Centre of Faculty of Technology and Metallurgy, University of Belgrade, 11000 Belgrade, Serbia; mmarkovic@tmf.bg.ac.rs (M.M.); vpanic@tmf.bg.ac.rs (V.P.); pspasojevic@tmf.bg.ac.rs (P.S.); bisailovic@tmf.bg.ac.rs (B.B.)
[2] Faculty of Technical Sciences, University of Kragujevac, 32000 Cacak, Serbia
[3] Innovative Centre of Faculty of Chemistry, University of Belgrade, 11000 Belgrade, Serbia; smilica84@gmail.com
[4] Faculty of Technology and Metallurgy, University of Belgrade, 11000 Belgrade, Serbia; sseslija@tmf.bg.ac.rs
* Correspondence: katarina.antic@tmf.bg.ac.rs

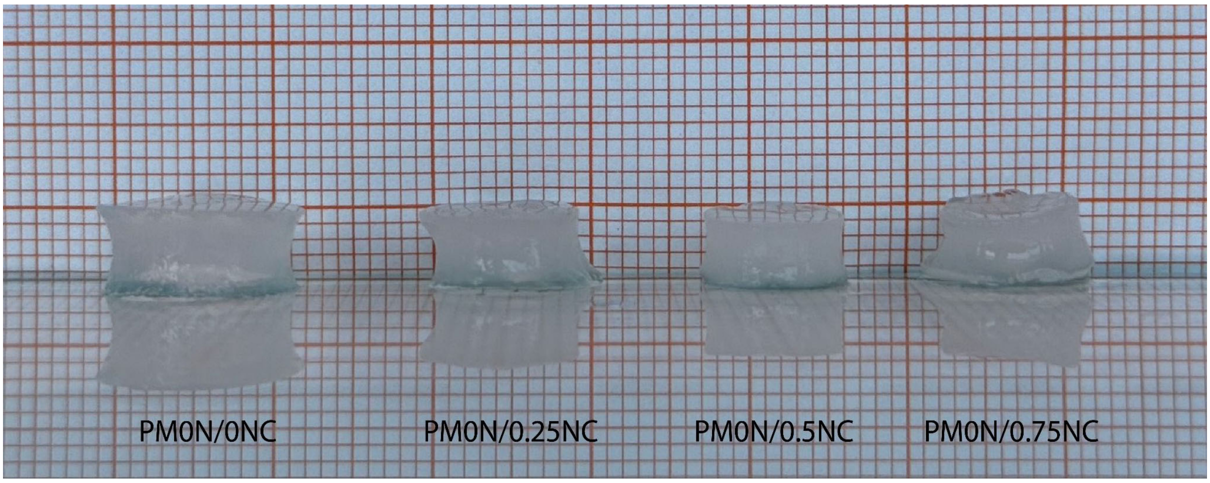

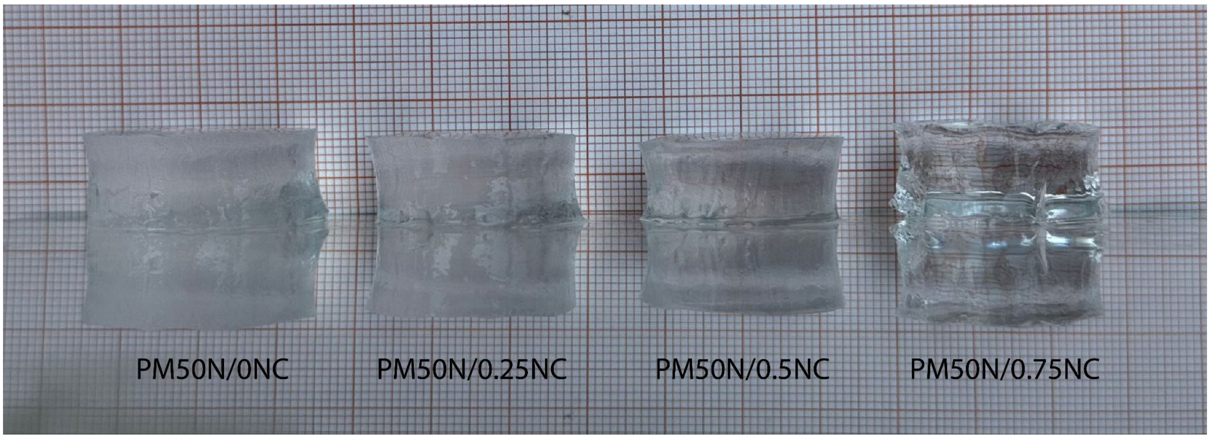

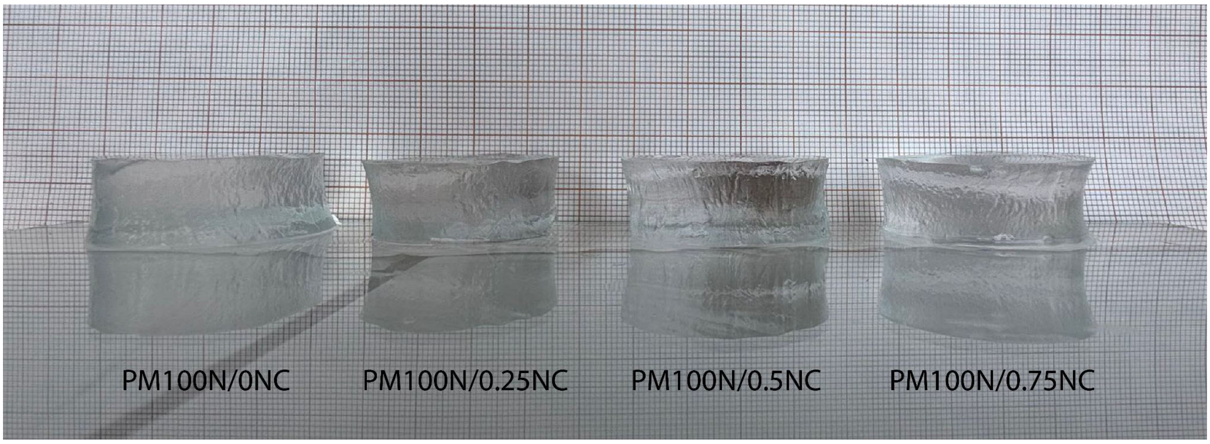

Figure S1. Images of PM0N/xNC, PM50N/xNC and PM100N/xNC, x = 0, 0.25, 0.5 and 0.75, hydrogels in the equilibrium state after swelling in distilled water at 25°C

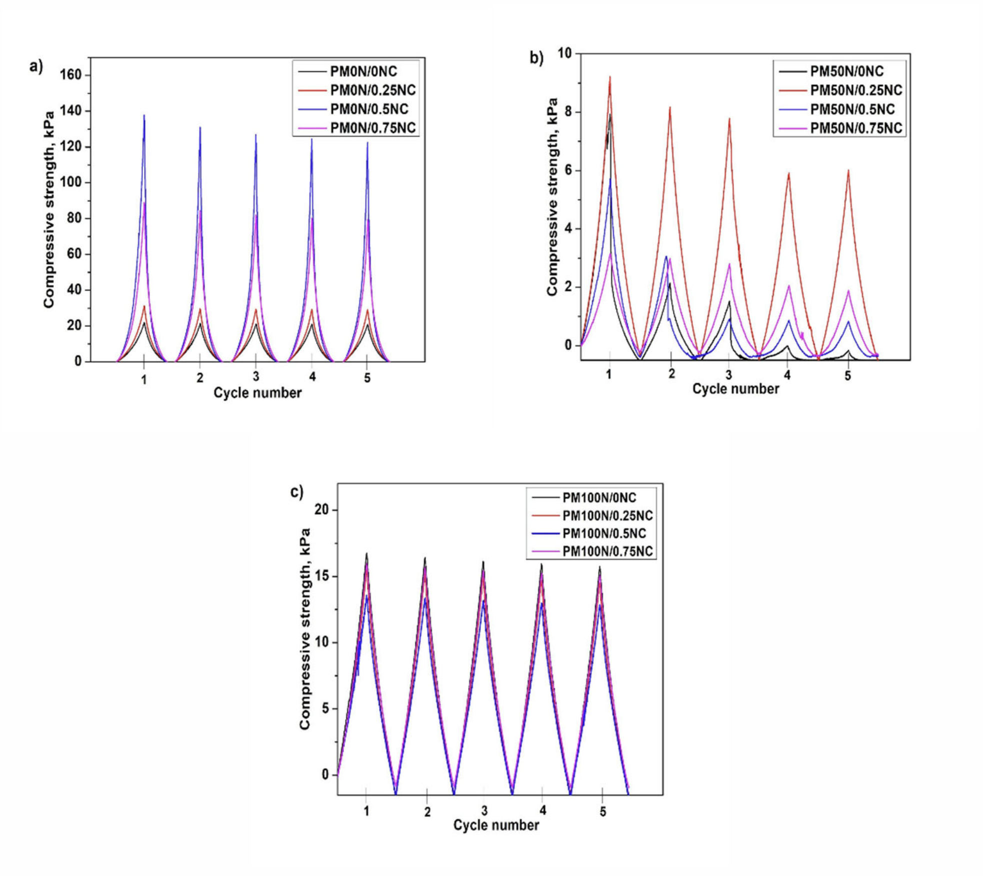

Figure S2. Compressive stress vs. cycle number for: a) PM0N/xNC, b) PM50N/xNC, and c) PM100N/xNC

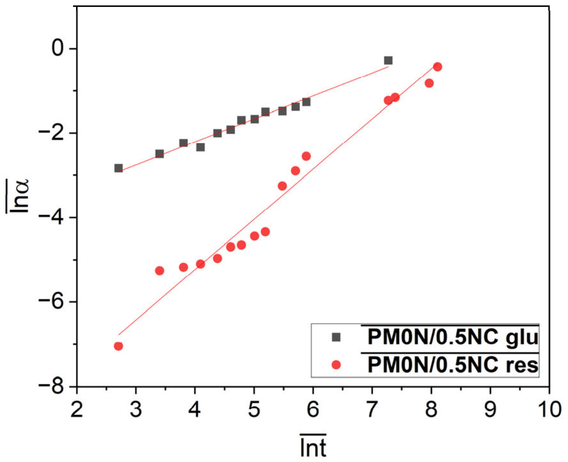

Figure S3. Determination of the release kinetics parameters from K-P model

Table S1. Comparison on swelling degree, compressive strength, and release profiles in recent key studies

System / Material	Swelling Degree	Compressive Strength / Modulus	Release Profile (kinetics, % released over time)	Released Substance / Model Drug	Reference
PMA–Gelatin (physically crosslinked)	/	Up to 24.81 MPa at 90% strain	Not specified	-	[1]
MAA/HEMA with nanocellulose (MHNC2B)	90.6% – 134.7% depending on NC content	/	Deswelling equilibrium ~95 min; >70% water release	Water (as deswelling model	[2]
Chitosan-gallic acid / methacrylic anhydride composite hydrogel	/	225 kPa	/	/	[3]
Poly(methacrylic acid) hydrogels under field	(depends on field)	/	Swelling kinetics described by Peppas model; field-dependent swelling behavio	/	[4]

SA-g-poly(MAA-co-AAm) / MMT nanocomposite hydrogel	/	/	Adsorption / release kinetics following pseudo-second-order model	Malachite Green (dye model)	[5]
Guar gum oleate-graft-poly(methacrylic acid) (GGO-g-PMAc) hydrogel	Max swelling at pH 7.4 (cca. 50 g/g); low swelling at pH 1.2 (cca. 10 g/g)	/	At ph 7.4 and 1.2; Controlled release in colon (pH-sensitive) Simulation of real GI microenvironment, the sequential drug release experiments in pH 1.2, 7.4 and 6.8 buffer media at 37 ◦C Ritger–Peppas release model	Ibuprofen	[6]

Table S2. Gel fraction

	Gel fraction, %		
Sample	0%N	50%N	100%N
PMXN/0NC	80.15	78.73	58.39
PMXN/0.25NC	80.84	82.82	59.25
PMXN/0.5NC	71.98	79.05	47.21
PMXN/0.75NC	86.36	75.14	52.69

Table S3. Peppas kinetic parameters for the swelling in pH5.5

	**0N**		**50N**		**100N**	
**NC**	**k/ min^{-1}**	**n**	**k/ min^{-1}**	**n**	**k/ min^{-1}**	**n**
0	0.015	0.63	0.089	0.41	0.054	0.50
0.25	0.018	0.62	0.21	0.28	0.10	0.38
0.50	0.014	0.64	0.37	0.17	0.11	0.39
0.75	0.023	0.58	0.23	0.22	0.092	0.36

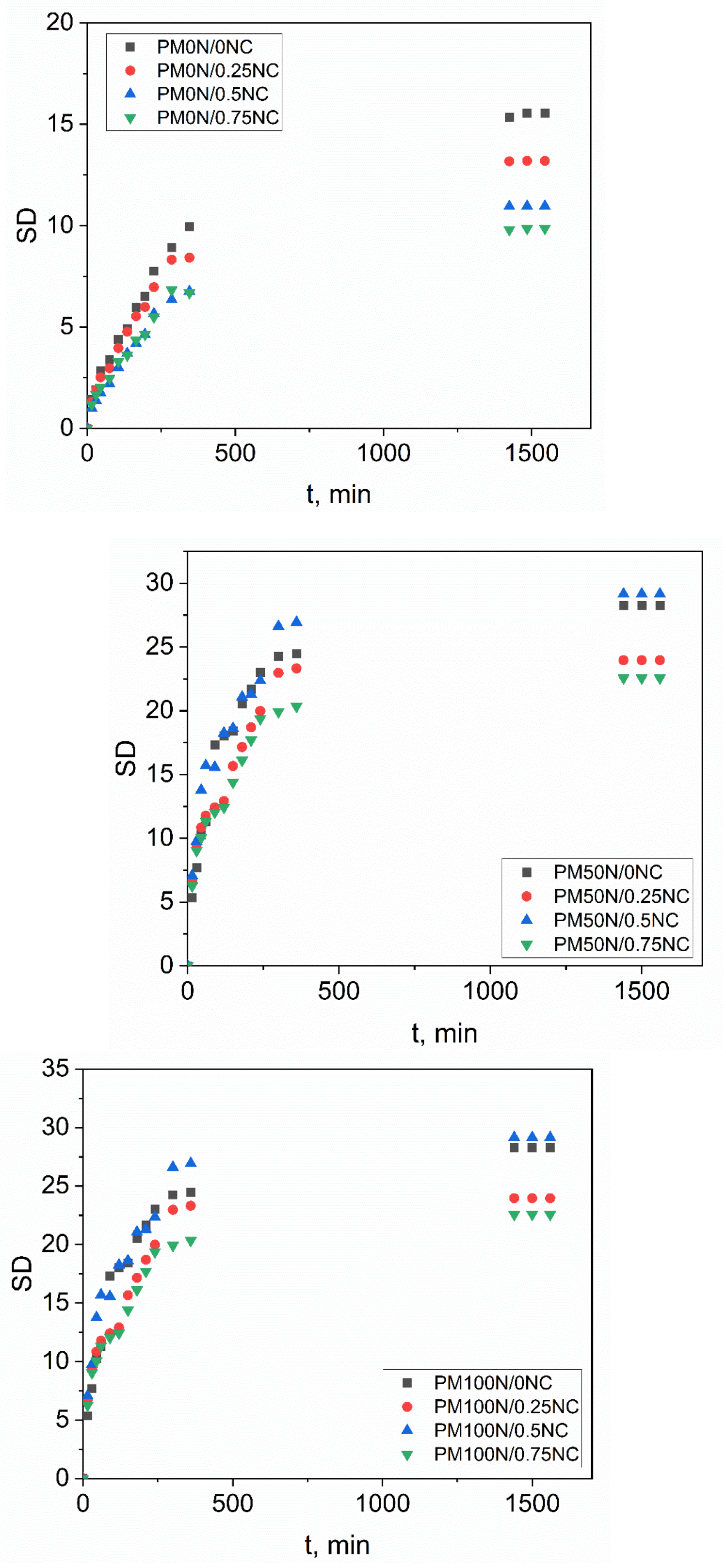

Figure S4. Swelling curves in pH5.5

Swelling in pH 5.5 buffer was clearly different compared to swelling in distilled water. Samples that swollen the fastest in water, 0N series, had the lower values of swelling rate parameter k in buffer, while the swelling process was notably accelerated for 50% and 100% neutralized samples.

The swelling behavior of the hydrogels in pH 5.5 buffer, described by the Peppas model parameters (k and n), reflects the interplay between the degree of PMAA neutralization, NC content, and the ionization state of PMAA carboxylic groups. Given that the buffer pH is slightly above the pKa of PMAA (~4.6), a considerable proportion of carboxyl groups are ionized in the 50N and especially 100N samples, resulting in electrostatic repulsion and network expansion. At 0% neutralization, PMAA remains largely in the –COOH form, leading to compact networks stabilized by hydrogen bonding. These samples show low swelling rates (k = 0.014–0.023 min^{-1}) and n values around 0.6, indicative of anomalous transport governed by both water diffusion and polymer relaxation. At 50% neutralization, swelling is significantly faster (k up to 0.37 min^{-1} at NC = 0.50), but n values decrease to 0.17–0.28, suggesting a predominantly Fickian diffusion mechanism. This behavior likely results from increased charge repulsion promoting rapid water uptake, while still maintaining some hydrogen bonding and interpolymer interactions that limit relaxation. In 100N samples, full ionization leads to moderate swelling rates (k = 0.054–0.11 min^{-1}), slightly lower than in 50N, with n values from 0.36 to 0.50, indicating diffusion-controlled swelling with limited structural relaxation. Importantly, despite these moderate swelling parameters in buffer, the 50N and 100N samples exhibited significantly higher swelling in distilled water, where the lack of ionic species reduces charge screening and allows stronger electrostatic repulsion, further expanding the network. This contrast highlights the effect of medium composition on swelling behavior: while electrostatic forces drive expansion in neutralized systems, the presence of buffer ions and lower pH in pH 5.5 medium mitigates these effects. Additionally, NC content strongly influences swelling at 50N, with maximum swelling observed at intermediate NC = 0.50mas%, followed by a decline at higher NC levels likely due to increased network density. In contrast, swelling parameters in 0N and 100N samples show less sensitivity to NC content, suggesting a more stable internal structure. Overall, the swelling behavior is highly tunable through neutralization and polymer composition, and the distinct response in buffer versus distilled water emphasizes the importance of environmental conditions when designing hydrogel systems for controlled drug delivery.

1. Ugrinovic, V., et al., *Physically Crosslinked Poly(methacrylic acid)/Gelatin Hydrogels with Excellent Fatigue Resistance and Shape Memory Properties.* Gels, 2024. **10**(7): p. 444.
2. Rabin Bera, A.D., Arpita Mandal, Arup Mandala and Debabrata Chakrabarty, *Modifying influences of micro crystalline and nanocellulose on the gelling characteristics of poly(methacrylic acid-co-2-hydroxyethylmethacrylate)* RCS Advances, 2016. **6**: p. 12616-12626
3. Tianfei Guo, W.S., Jun Li, Siqin Huang, Guixiang Hou, *Preparation and Performance of Chitosan-gallic acid/Methacrylic Anhydride Composite Antibacterial Hydrogel* Academic Journal of Science and Technology, 2024. **12**(1).
4. Jovanovic, J.D., et al., *Field Responsive Swelling of Poly(Methacrylic Acid) Hydrogel—Isothermal Kinetic Analysis.* Polymers, 2025. **17**(19): p. 2602.
5. Safarzadeh, H., Peighambardoust, S.J. & Peighambardoust, S.H., *Application of a novel sodium alginate-graft-poly(methacrylic acid-co-acrylamide)/montmorillonite nanocomposite hydrogel for removal of malachite green from wastewater.* Journal of Polymer Research, 2023. **30**(157).
6. D. Sathya Seeli, M.P., *Guar gum oleate-graft-poly(methacrylic acid) hydrogel as a colon-specific controlled drug delivery carrier.* Carbohydrate Polymers, 2017. **158**: p. 51-57.
